# New Potential Therapeutic Approach for the Treatment of B-Cell Malignancies Using Chlorambucil/Hydroxychloroquine-Loaded Anti-CD20 Nanoparticles

**DOI:** 10.1371/journal.pone.0074216

**Published:** 2013-09-30

**Authors:** Nelly Mezzaroba, Sonia Zorzet, Erika Secco, Stefania Biffi, Claudio Tripodo, Marco Calvaruso, Ramiro Mendoza-Maldonado, Sara Capolla, Marilena Granzotto, Ruben Spretz, Gustavo Larsen, Sandra Noriega, Marianna Lucafò, Eduardo Mansilla, Chiara Garrovo, Gustavo H. Marín, Gabriele Baj, Valter Gattei, Gabriele Pozzato, Luis Núñez, Paolo Macor

**Affiliations:** 1 Dept. of Life Sciences, University of Trieste, Trieste, Italy; 2 Institute for Maternal and Child Health – IRCCS “Burlo Garofolo”, Trieste, Italy; 3 Department of Human Pathology, University of Palermo, Palermo, Italy; 4 Molecular Medicine Laboratory, International Centre for Genetic Engineering and Biotechnology, Trieste, Italy; 5 LNK Chemsolutions LLC, Lincoln, Nebraska, United States of America; 6 Bio-Target, Chicago, Illinois, United States of America; 7 Centro Único Coordinador de Ablación e Implante Provincia de Buenos Aires, Ministry of Health, La Plata, Buenos Aires, Argentina; 8 Optical Imaging Laboratory, Cluster in BioMedicine, Trieste, Italy; 9 Clinical and Experimental Onco-Hematology Unit, Centro di Riferimento Oncologico, Aviano, Italy; 10 Dipartimento Universitario Clinico di Scienze mediche, Chirurgiche e della Salute, University of Trieste, Trieste, Italy; 11 University of Chicago, Chicago, Illinois, United States of America; European Institute of Oncology, Italy

## Abstract

Current B-cell disorder treatments take advantage of dose-intensive chemotherapy regimens and immunotherapy via use of monoclonal antibodies. Unfortunately, they may lead to insufficient tumor distribution of therapeutic agents, and often cause adverse effects on patients. In this contribution, we propose a novel therapeutic approach in which relatively high doses of Hydroxychloroquine and Chlorambucil were loaded into biodegradable nanoparticles coated with an anti-CD20 antibody. We demonstrate their ability to effectively target and internalize in tumor B-cells. Moreover, these nanoparticles were able to kill not only p53 mutated/deleted lymphoma cell lines expressing a low amount of CD20, but also circulating primary cells purified from chronic lymphocitic leukemia patients. Their safety was demonstrated in healthy mice, and their therapeutic effects in a new model of Burkitt's lymphoma. The latter serves as a prototype of an aggressive lympho-proliferative disease. In vitro and in vivo data showed the ability of anti-CD20 nanoparticles loaded with Hydroxychloroquine and Chlorambucil to increase tumor cell killing in comparison to free cytotoxic agents or Rituximab. These results shed light on the potential of anti-CD20 nanoparticles carrying Hydroxychloroquine and Chlorambucil for controlling a disseminated model of aggressive lymphoma, and lend credence to the idea of adopting this therapeutic approach for the treatment of B-cell disorders.

## Introduction

B-cell malignancies are a heterogeneous group of clinical conditions with highly variable clinical courses that span between indolent diseases like the chronic lymphocytic leukemia (CLL) and highly aggressive lymphoproliferative disorders, like Burkitt lymphoma (BL) [1], [2], [3], [4]. B-cell tumor treatments include dose-intensive chemotherapy regimens and immunotherapy via monoclonal antibodies (mAbs) [5]. Despite the promising survival rates, these intensive multi-agent treatments display a high degree of toxicity, and a significant percentage of patients are also unresponsive [6], [7], [8]. Several limitations have been described to explain refractory/relapse patients. In particular, genetic modification in specific onco- or oncosuppressor genes, such as p53 [9], is associated with unsuccessful chemotherapeutic regimens. In contrast, antibody-based immunotherapy has little side effects but its efficacy is mainly driven by the expression of sufficient amounts of tumor-associated antigen on the neoplastic cell surface [10].

In recent years, nanotechnology has attracted significant interest from oncologists given its potential to offer a new paradigm to overcome complex therapeutic targeting [11], [12], [13]. Nanoparticles made with biodegradable biopolymers (BNPs) as carrier material have been extensively investigated for sustained and controlled delivery of imaging and therapeutic agents with high efficacy and minor side effects [14], [15], [16], [17], [18], [19]. Targeted delivery of nanoparticles can be achieved by attaching specific ligands or antibodies onto the nanoparticle surface [20], [21], [22], [23], [24], [25].

In this study, we developed a novel therapeutic approach in which the efficacy of high-dose chemotherapy is a consequence of the specificity and low side effects of antibody-based therapy. This approach is based on biodegradable nanoparticles coated with an antibody to target cells, and loaded with Hydroxychloroquine (HCQ) and Chlorambucil (CLB) to specifically kill the cancer cells. For the first time, we demonstrate the ability of a certain class of nanoparticles to kill p53 mutated/deleted leukemia/lymphoma cells expressing a low amount of CD20, and their safety and therapeutic effects in a BL model, as an aggressive lymphoprolipherative disease prototype.

## Materials and Methods

### Cells, antibodies and sera

BL cell lines (BJAB and Raji) were cultured in RPMI-1640 medium (Sigma-Aldrich, Milan, Italy) supplemented with 10% fetal calf serum (FCS; Gibco, Invitrogen, Milan, Italy).

Heparinized peripheral blood samples were obtained after written informed consent from B-CLL untreated patients at the Maggiore Hospital in Trieste. Patients provided informed consent in accordance with IRB requirements and The Declaration of Helsinki. The study was approved by the IRB of the CRO (IRCCS) of Aviano (IRB-06–2010). The mononuclear cell fractions were isolated by centrifugation on Ficoll-Hypaque (GE Healthcare, Milan, Italy) density gradients.

BJAB cells suspended in serum-free RPMI-1640 medium were stained with Vybrant^TM^ DiD cell-labeling solution (GE Healthcare) as previously reported [26].

The anti-CD20 chimeric mAb Rituximab (Roche, Milan, Italy) was obtained from the clinical facilities (University of Trieste, Italy). The mAb CD20 was secured from BioLegend (San Diego, CA), and anti-PARP1 antibody was obtained from Bethyl Laboratories. The anti-LC3 and anti-α-tubulin mAb were from Sigma-Aldrich, and anti-p62 mAb was from Becton Dickinson (Milan, Italy). For the immunophenotypical characterization studies, anti-human-CD20 (clone L26, Novacastra), anti-human-BCL6 (clone P1F1, Novacastra) and anti-human-Ki67 (clone Mib-1, Dako, Denmark) mAbs were used.

Human sera from AB Rh+ blood donors were kindly provided by the Blood Transfusion Center (Trieste, Italy) as a source of complement (NHS – normal human serum).

### BNPs preparation

Chemicals used for BNPs preparation were reagent grade or better. Some of the chemicals purchased were: polyethylene glycol (PEG) from Nektar, San Carlos, CA; Hydroxychloroquine sulfate (HCQ) from ACROS, Gel Belgium; Chlorambucil (CLB) from Sigma Aldrich, St Louis, MO. BNPs, based on carboxylic acid terminated biodegradable polymers (PLA-b-PEG-COOH and PCL-COOH), were produced with average diameter of 250 nm, as measured by dynamic light scattering (data not shown). The particles were fabricated under class 100 clean room conditions by implementing Bio-Target's technology at LNK Chemsolutions LLC laboratories [25].

All BNPs were resuspended at the time of use in PBS buffer (pH = 7.4) with 10% BSA at a final total concentration of 900µg/mL.

### Animals

Female SCID mice (4–6 weeks of age) were purchased from Charles River (Milan, Italy) and maintained under pathogen-free conditions. C57/BL mice were obtained from the Animal House of the University of Trieste.

All the experimental procedures involving animals were done in compliance with the guidelines of the European (86/609/EEC) and the Italian (D.L.116/92) laws, and were approved by both the Italian Ministry of Health and the Administration of the University Animal House (Prot. 42/2012).

### FACS analysis

BJAB cells were labeled using anti-CD20 mAb at 10 μg/mL and high and low CD20 expressing BJAB cells were isolated by cell sorting on a BD FACS Aria II flow cytometer and CELLQuest software.

### Electron microscopy analysis

Samples were treated as previously described [27]. Ultrathin sections were prepared with an Ultratome III (LKB, Pharmacia) and observed by transmission electron microscopy (TEM; EM208; Philips, Eindhoven, The Netherlands). Micrographs were taken with a Morada Camera (Olympus Soft Imaging Solutions, Munster, Germany).

### Cell viability, apoptosis and autophagy

To investigate the ability of BNPs to affect cell viability, BJAB cells (2×10^5^) were incubated with BNPs for 48 hours (in a humidified 37°C, 5% CO_2_ incubator). The number of residual viable cells was determined via the MTT assay, and the percentage of dead cells was calculated according to the formula, %D =  [(test release – spontaneous release)/(total release – spontaneous release)] ×100.

Apoptosis was measured using FITC-labeled recombinant human Annexin V assay (Apoptosis detection kit, Immunostep, Spain) following the manufacturer's instructions. For each measurement 30,000 events were acquired with a standard FACSCalibur (Becton Dickinson, San Jose, CA) flow cytometer and analysis of data were performed with CellQuest (Becton Dickinson). PARP-1, LC-3 and p62 activation were evaluated via immunoblotting in order to show apoptosis and autophagy [28].

### Complement-mediated lysis

A previously described procedure of Complement Dependent Cytotoxicity (CDC) with some modifications was used to evaluate the effect of Rituximab on complement-mediated killing of tumor B-cells [29]. The number of residual viable cells was estimated using the MTT assay.

### Mouse Model of B-Lymphoma

SCID mice were inoculated i.p. on the right flank with 2×10^6^ BJAB cells and examined twice weekly up to 120 days for signs of sickness.

Mice receiving the labeled cells were analyzed *in vivo* every day and *ex vivo* on days 4, 7, 21 and 25 following cell injection using the small-animal time-domain Optix MX2 preclinical NIRF-imager (Advanced Research Technologies, Montreal, CA), as previously described [26]. At the end of the *in vivo* evaluation, the animals were sacrificed to perform *ex vivo* optical imaging analyses. The peritoneal mass and other organs of interest such as liver, kidney, spleen, heart, lung, lymph nodes and thigh bones were collected, washed in PBS and analyzed by the eXplore Optix preclinical imager (GE Healthcare).

### Histopathological and Immunohistochemical analysis

Tissue specimens from peritoneal mass and other organs obtained from lymphoma-bearing mice at necropsy were treated as previously described [29]. Snap-frozen samples embedded in OCT Compound Embedding Medium (Diagnostic Division, Miles, Inc.) were analyzed using fluorescence microscope Leica DM2000 (Leica, Milan, Italy).

### Statistical Analysis

The data were expressed as mean ± SD and analyzed for statistical significance by the two-tailed Student's t test to compare two paired groups of data. The Kaplan-Meier product-limit method was used to estimate survival curves and the log-rank test was adopted to compare different groups of mice.

## Results

### Anti-CD20 BNPs Target Tumor B-cells

Four different types of nanoparticles named BNP0, BNP1, BNP2 and BNP3 were initially prepared as described in Figure S1. BNP0 were prepared only with PLA-b-PEG-COOH and PCL-COOH as polymeric carrier. BNP1 were prepared with the anti-human CD20 on the surface. BNP2 particles were produced by encapsulation of HCQ sulfate and CLB inside their core, and modified by Rituximab on the surface. BNP3 were prepared as BNP2 without anti-CD20 antibody on the surface.

**Figure 1:**
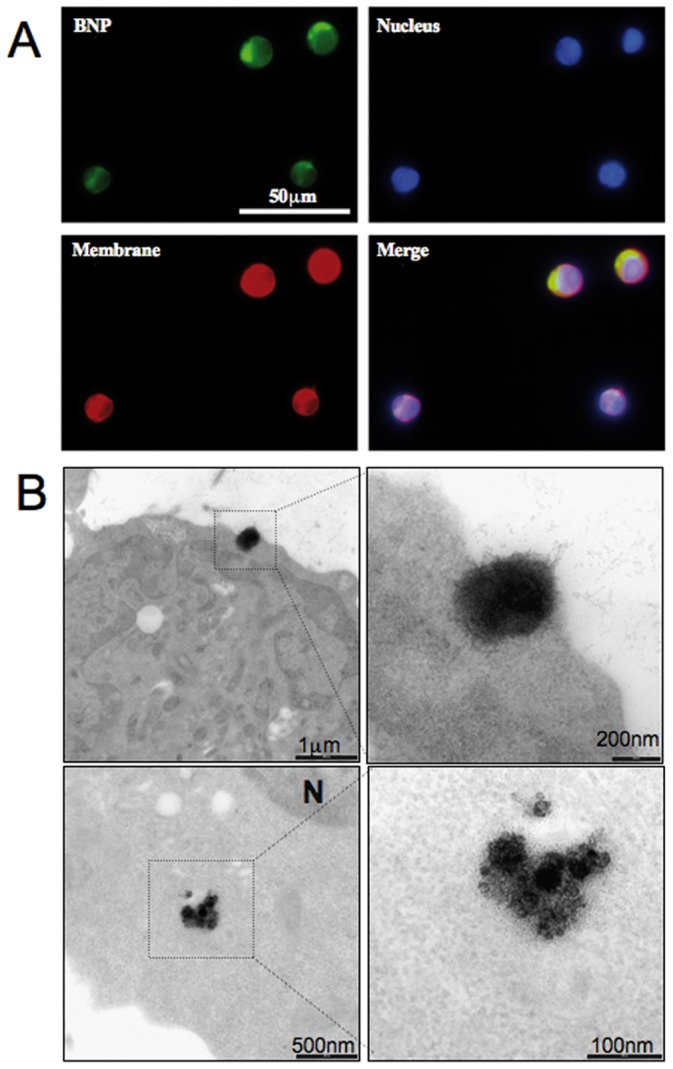
Interacion between anti-CD20 BNPs and B-cells. A) Binding of anti-CD20 BNP to BJAB cells. BJAB cells were labeled with FAST-DiO (membrane-red) and DAPI (Nucleous-blue) and incubated with FITC-labeled BNP1 (Green) for 1 hour at 37°C; samples were analyzed using confocal microscopy. Original magnification 200×. B) Internalization of anti-CD20 BNP to BJAB cells. Bjab cells were incubated with BNPB for 1h and analyzed by TEM.

The characterization of BNPs started by evaluating their ability to bind to lymphoma cells. To this aim, BNP0 and BNP1 were labeled with FITC and added to the BL cell line BJAB. The BNP1 system was able to target BJAB cells in a dose- and time-dependent manner. The maximal uptake was measured after an incubation of 1 hour using 2 μL of BNP1, when all the cells appeared tagged by BNP1 (Figure 1A). The BNP0 particles did not evidence specific binding in one hour.

TEM studies were performed, following BNP migration into tumor B-cells. To this end, two different types of BNPs (named BNPA and BNPB) were prepared, as shown in Figure S1. BNPA were produced by encapsulation of gadopentetate dimeglumine (Magnevist®, Bayer HealthCare Pharmaceuticals Inc) while in BNPB the anti-CD20 antibody was attached to the surface of BNPA. BJAB cells were incubated with BNPA and BNPB and then analyzed by TEM, exploiting the presence of Gd in the particles. Images showed the binding of BNPB and their interaction with the cell surface; the nanoparticles accumulated in the cytoplasma of tumor cells but their internalization showed no evidence of endocytotic vesicles involvement (Figure 1B). A limited data set on the effect on BNPA in BJAB cells at different times were documented (data not shown).

### BNP2 Induce Tumor B-Cell Cytotoxicity

HCQ and CLB were chosen as therapeutic drugs. BJAB cells incubated with HCQ (5.4 μg in 2 μL, 80 μM) resulted in 82% of tumor cell death after 48 hours. Under the same conditions, CLB (5.4 μg in 2 μL, 89 μM) is able to kill about 38% of BJAB cells. As observed in other p53 mutated cells, like Raji, BJAB is quite resistant to CLB and other classical chemotherapeutic agents such as anthracyclines and purine analogues; in fact, the same doses of fludarabine and doxorubicin kill 42% and 37% of cells, respectively. Our data indicates that only CLB shows an additive effect with HCQ, causing 92% of killing (HCQ+CLB vs. HCQ: p<0.01), and these drugs were loaded in BNP2 and BNP3.

**Figure 2:**
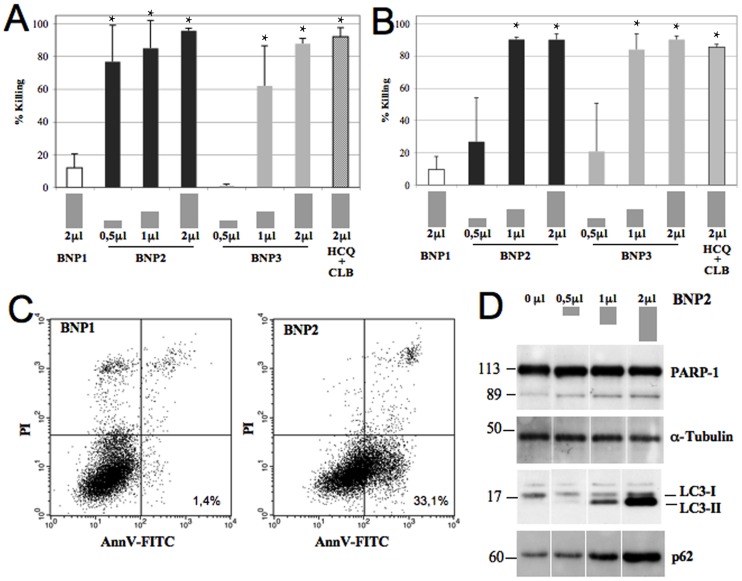
*In vitro* characterization of the cytotoxic effect of BNP2. BJAB (A) and Raji (B) cells were incubated with 0.5, 1 and 2 μL of BNPs or HCQ+CLB for 48 hours at 37°C and residual viable cells were measured. Data are expressed as mean ± SD. *: p<0.01 vs BNP1. C) BJAB cells wer incubated with 1 μL of BNPs for only 16 hours at 37°C and apoptotic cells were analyzed using AnnexinV/PI test. D) Western blot analysis of activated PARP-1, LC3 and p62 from cell lysates obtained from BJAB cells incubated with 0, 0.5, 1 and 2 μL of BNP2.

In order to evaluate the cytotoxic effect of BNPs, BJAB or Raji lymphoma cells were incubated with different amount of BNP0, BNP1, BNP2 and BNP3 and residual viable cells were measured. BNP2 and BNP3 were able to induce cell cytotoxicity in a dose-dependent manner while BNP0 and BNP1 were practically ineffective. All tumor cells were killed in 48 hours using only 2 μL of BNP2 or BNP3 containing 5.4 μg of HCQ and CLB (Figure 2A–B). This result is due to the pro-apoptotic effect induced by the chemotherapeutic drugs. In fact, more than 30% of tumor cells incubated for only 16 hours with 1 μl of BNP2 showed the apoptotic profile in an Annexin V/PI test (Figure 2C). No molecular studies were possible using 2 μL of BNP2 for 48 hours.

The poly (ADP-ribose) polymerase (PARP-1), a 113 kDa nuclear enzyme, is cleaved in fragments of 89 and 24 KDa during apoptosis and is a hallmark of the process. In fact, the PARP-1 cleavage was detected in BJAB treated with a different amount of BNP2 particles for only 16 hours by western blot assay of cell lysate (Figure 2D).

The presence of HCQ in BNP2 could also induce an impaired autophagy [30]. It has been demonstrated that the LC3 protein during autophagy is processed to a cytosolic version (LC3-I, 18 KDa), and then converted to a lipidized form (LC3-II, 16 KDa) that stably associates with the membrane of phagophores, autophagosomes or autophagolysosomes [31]. LC3-II is commonly used as a marker of autophagosome accumulation, caused either by induction of their formation, or by inhibition of fusion with lysosomes. The effect of HCQ in nanoparticles is clearly demonstrated by analyzing LC3 in cell lysate of BJAB incubated with BNP2. Cells treated with a different amount of nanoparticles showed a dose-dependent accumulation of LC3-II (Figure 2D). p62 is a protein recruited to the autophagosomal membrane, and represents another marker of autophagosome accumulation. The effect of HCQ was confirmed by analyzing the presence of p62 protein in the same samples. The amount of p62 was again increased in a dose-dependent manner after the treatment of lymphoma cells with BNP2 (Figure 2D).

### Comparison Between BNP2 and Rituximab Cytotoxic Effects

The anti-CD20 chimeric antibody Rituximab mainly works by activating the complement system, and antibody-dependent cell cytotoxicity (ADCC). In contrast, a very low killing effect is due to its ability to activate apoptotic pathways. For this reason, we have compared the killing of BJAB cells expressing high and low amounts of CD20 induced by a saturating concentration of Rituximab (complement-dependent killing), or by BNP2 (apoptosis). BJAB cells were analyzed using anti-CD20 antibodies (Mean Fluorescence Intensity-MFI: 316) and sorted in order to obtain two different populations expressing high (MFI: 602) and low (MFI: 103) amounts of CD20 on their surface. Rituximab was able to kill up to 22% of BJAB [32] or high-CD20 BJAB, but it was unable to lyse low-CD20 BJAB. On the other hand, BNP2 killed 92% of BJAB or 93% of high-CD20 BJAB and maintained the capacity to induce cell death in 83% of low-CD20 tumor cells (Table 1).

**Table 1:** Comparison between BNP2 and Rituximab effects.

	CD20	BNP2	RITUXIMAB
	(MFI)	(% killing)	(% killing)
**BJAB**	316,8	92,0	18,9
**BJAB-High**	602,9	93,2	20,6
**BJAB-low**	103,1	83,1	0,6
**Pz1**	73,6	77,4	0,0
**Pz2**	22,2	81,7	8,9
**Pz3**	25,3	55,1	9,9
**Pz4**	41,4	44,9	18,0
**Pz5**	64,5	47,9	21,8
**Median**	41,4	55,1	9,9

BJAB cells were sorted to obtain High-CD20 and Low-CD20 cells. Mononuclear cells were purified from untreated CLL patients. Cells were analyzed for CD20 expression (MFI-mean fluorescence intensity) and then incubated with BNP2 for 48 hours to induce apoptosis. Cells were also incubated with Rituximab + NHS as a source of Complement for 1 h to induce CDC. Residual viable cells were measure using MTT assay.

Circulating tumor B-cells in a CLL patient are also characterized by the expression of a low amount of CD20 on the cell surface, as we documented in purified cells. The cytotoxic effect of Rituximab on these cells ranged between 0 and 22%, with a median value of 9.9%. Conversely, BNP2 killed up to 82% of tumor B-cell, with a median value of 55.1% (BNP2 vs. Rituximab: p<0.0001) (Table 1).

### BNPs Show a Safe Toxicological Profile

**Figure 3:**
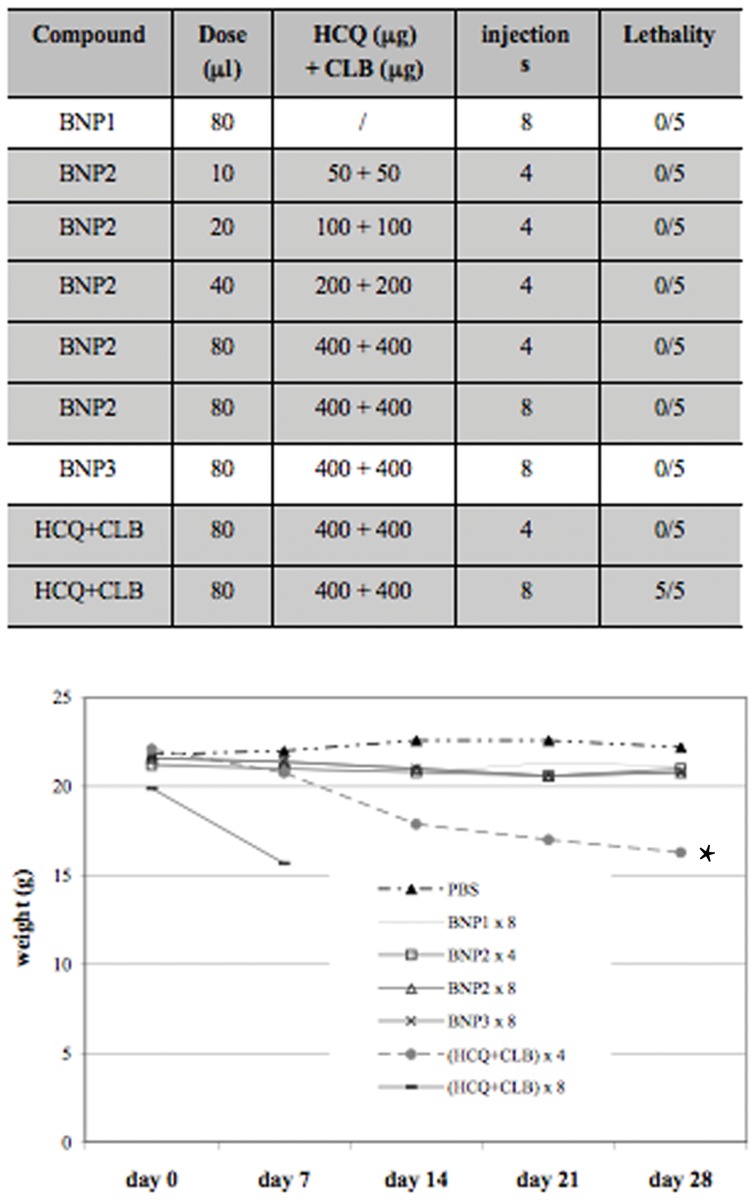
Toxicological studies. C57/BL mice received BNP1, BNP2, BNP3 and HCQ+CLB at different doses. A) Animal survival and B) total body weight was measured for 28 days to evaluate toxicity of the treatments. *: p<0.001 vs PBS.

The toxic effects induced by the intra-peritoneal injection of BNPs were evaluated in C57/BL mice by analyzing total body weight and survival. Groups of five animals received different doses of BNP1, BNP2, BNP3 or free HCQ+CLB and were followed for 28 days. Only the mice receiving 8 times the mixture of free HCQ+CLB died in this experiment (Figure 3A), but the analysis of body weight showed a significant toxicity also in mice receiving 4 i.p. injections of the two drugs (untreated vs. HCQ+CLB ×4: p<0.001) (Figure 3B). BNPs appear to have a very safe toxicological profile, without significant loss of body weight in all treated mice (Figure 3B). These data were also confirmed by histological analysis of mice tissues; in fact, all samples collected from BNPs-treated animals maintained a normal morphology (data not shown).

### Development of a Burkitt Lymphoma Model Using BJAB Cells

**Figure 4:**
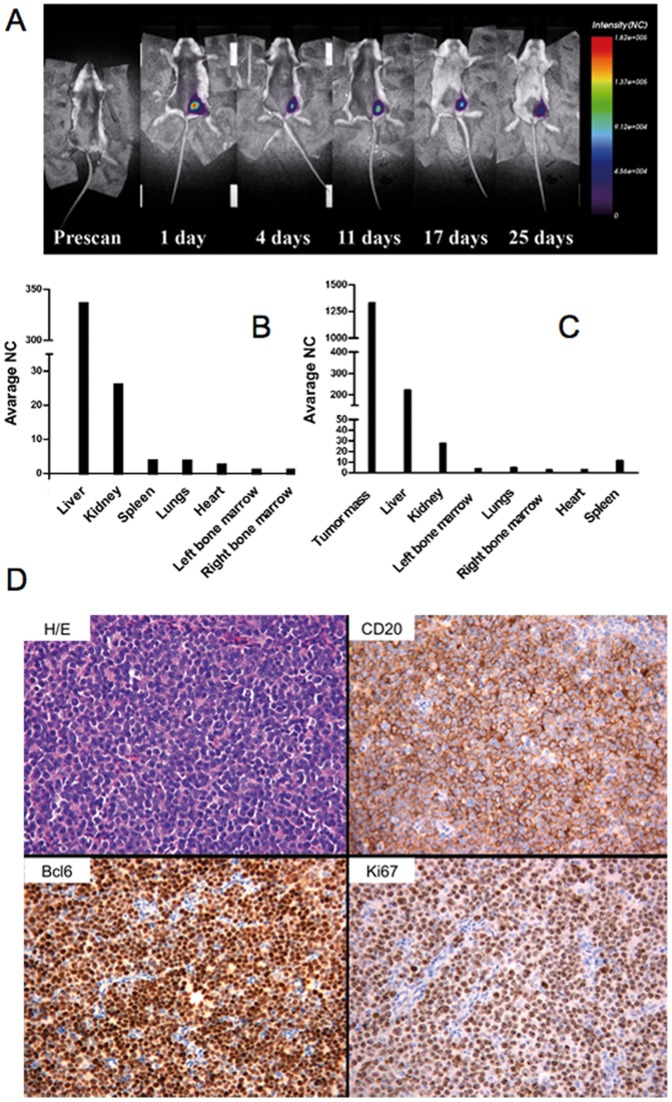
Characterization of Burkitt model in SCID mice. Labeled BJAB (2×10^6^ cells) were injected i.p. in SCID mice and fluorescence intensity emissions were acquired *in vivo* for 25 days. (A) Whole body scans at indicated post-injection time are reported. (B) To evaluate the dissemination to multiple organs by *ex vivo* analysis, mice were injected with labeled cells and they were sacrificed 7 days after the injection. (C) The same *ex vivo* analysis was performed on mice 25 days after the BJAB injection, when the tumor mass was already developed. NC =  Normalized Count. D) Peritoneal tumor mass displays a solid cohesive pattern of growth with round small/medium sized elements and a high number of mitotic and apoptotic figures (Hematoxilin and Eosin, original magnification 200×). Neoplastic cells show strong immunoreactivity to CD20 (anti-CD20 immunostaining, Strept-ABC method, original magnification 200×) and Bcl-6 (anti-Bcl-6 immunostaining, Strept-ABC, original magnification 200×). The high proliferation rate of neoplastic cells (nearly 100% of cells) is highlighted by Ki-67 immunostaining (anti-Ki-67, Strep-ABC method, original magnification 100×).

In order to characterize the potential *in vivo* effect of BNP2, we set up a human/mouse model of BL using BJAB cells in SCID mice. Tumor cell distribution was analyzed using near-infrared time-domain optical imaging; intra-peritoneal injection of DiD-labeled BJAB induced a fast tumor cell distribution, demonstrated already after four days for the presence of BJAB cells in different organs (Figure 4B). A tumor mass was grown at the site of injection in 20–25 days in all SCID mice (Figure 4A and C). Localization of tumor cells in liver, spleen, bone marrow, kidney and lung was further confirmed by confocal microscopy in sections of tissue samples obtained 7 days after cell injection (Figure S2), and was partially documented in SCID mice up to 25 days after inoculation of BJAB cells (data not shown). Figure 4D shows sections of a peritoneal tumor mass collected on day 25 from mice xenograft, stained with H&E and examined for the presence of lymphoma cells. Immunohistochemical analysis revealed strong membrane reactivity with antibody to human CD20, cytosolic presence of BCL-6 and Ki-67 as markers of human lymphoma cells (Figure 4D). The liver was involved in all the analyzed animals but BJAB cells were also detected in the bone marrow of 14 mice out of 15 (Figure S3). None of the other organs analyzed showed cell infiltration except for the spleen of 6 out 15 animals and the kidneys in two animals, but in both cases the organ involvement appeared to be “ab extrinseco”. All animals died between 50 and 70 days after tumor cell injection.

### BNP2 Demonstrates a Therapeutic Effect in a Disseminated Burkitt Lymphoma Model

**Figure 5:**
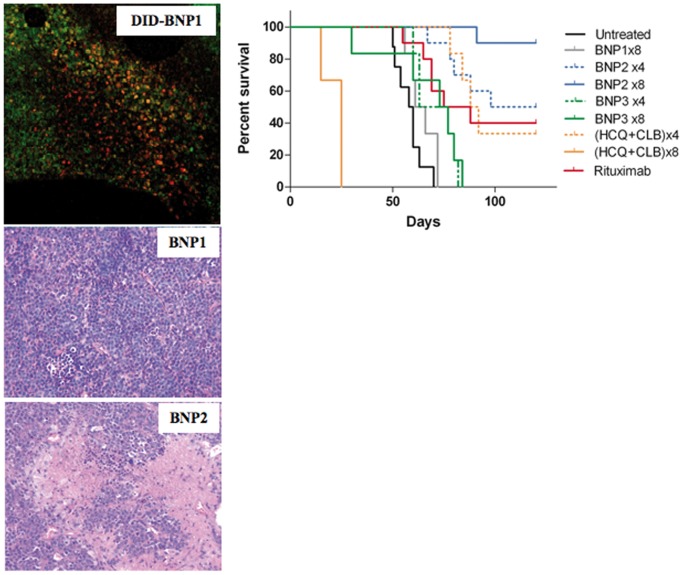
Therapeutic effect of BNPs, HCQ+CLB and Rituximab. A) SCID mice (n = 5 per group) received 2×10^6^ BJAB cells i.p.; Cy5.5 labeled-BNP1 or BNP2 (40 μL for 3 times in 5 days) were injected in tumor-bearing mice with a visible peritoneal tumor mass at day 25; the animals were sacrificed 7 days after the end of the treatment and the tumor masses were visualized by confocal microscopy and analyzed by H&E. Original magnification 200×. B) Survival curve. SCID mice (n = 7–10 per group) received 2×10^6^ BJAB cells i.p. and BNP1, BNP2, BNP3, HCQ+CLB or Rituximab as described in the results. P values. Untreated vs. BNP1: Not significant; Untreated vs. BNP2×4: p<0.0001; Untreated vs. BNP2×8: p<0.0001; Untreated vs. (HCQ+CLB)×4: p<0.0003; Untreated vs. Rituximab: p<0.0005; BNP2×4 vs. (HCQ+CLB) ×4: Not significant; BNP2×4 vs. Rituximab: Not significant; BNP2×8 vs. (HCQ+CLB) ×8: p<0.0001; BNP2×8 vs. Rituximab: p<0.0003; BNP3×8 vs BNP1: Not significant; BNP3×8 vs BNP2×4: p<0.0005; BNP3×8 vs BNP2×8: p<0.0001.

DID-labeled BNP1 and BNP2 were injected i.p. (40 μL for 3 times in 5 days) in five tumor-bearing mice, with a visible peritoneal tumor mass. These animals were sacrificed 7 days after the end of the treatment. The tumor masses were analyzed by confocal microscopy and H&E to detect the presence of nanoparticles and apoptotic/necrotic areas induced by BNPs activity. Figure 5 demonstrates the presence of DID-labeled anti-CD20 nanoparticles 7 days after their injection. Apoptotic/necrotic areas in tumor masses were documented in all animals receiving BNP2 while no cytotoxic effect was evident in BNP1-treated mice (figure 5).

All the *in vivo* data showed that BNP2 injection is safe, and is able to induce tumor cell death in a human/mouse model of BL. We then tested the efficacy of BNP2 in the treatment of the human/SCID model of BL (Figure 5). To this goal, BJAB cells were injected in SCID mice and divided into groups of 7–10 animals, and followed for 120 days.

Group 1 was kept untreated, and mice died within 50 to 70 days after tumor cell injection.

Group 2 received 8 injections of 80 μL of BNP1, but the treatment did not significantly increase their survival.

Groups 3 and 4 were challenged with the mixture HCQ+CLB (400 μg each) for 4 times in 8 days or 8 times in 17 days respectively. Eight injections of HCQ+CLB confirmed the data obtained with healthy mice, and all animals died during the treatment. Four i.p. injections of chemotherapeutic drugs were tolerated also by tumor-bearing mice, and caused a cytotoxic effect of tumor cells that induced 33% of mice survival (Untreated vs. (HCQ+CLB) ×4: p<0.0003).

Groups 5 received 80 μL of BNP2 (corresponding to 400 μg of each encapsulated chemotherapeutic agent targeted via anti-CD20 antibody) for 4 times in 8 days; this treatment rendered a survival curve similar to the profile obtained for group 3, but 50% of the mice were cured by this treatment (Untreated vs. BNP2×4: p<0.0001; BNP2×4 vs. (HCQ+CLB)×4: Not significant).

Group 6 received 80 μL of BNP2 for 8 times in 17 days. This treatment was effective in all the tumor-bearing mice and 90 days after tumor challenge 100% of animals were still alive. At the end of the experiment, only one mouse out of 10 died for the development of lymphoma and 90% of mice were cured (Untreated vs. BNP2×8: p<0.0001; BNP2×8 vs. (HCQ+CLB) ×8: p<0.0001), as subsequently demonstrated by immunohistochemistry analysis of all the organs of these animals (data not shown).

Groups 7 received 80 μL of nanoparticles without antibodies (BNP3) for 4 times, with an equal amount of chemotherapeutic agents in group 5 (BNP2) and group 3 (free agents). No significant therapeutic effect was observed.

Group 8 received 80 μL of BNP3 for 8 times in 17 days, containing the same amount of HCQ+CLB injected in group 6 and 4. Animals did not evidence toxicity but BNP3 has no significant therapeutic effects (BNP3×8 vs Untreated: Not significant; BNP3×8 vs BNP1: Not significant; BNP3×8 vs BNP2×4: p<0.0005; BNP3×8 vs BNP2×8: p<0.0001).

Rituximab (12.5 μg at days 4 and 11 [29]) was injected in animals of group 9 resulting in a 40% of mice survival after this treatment (Untreated vs. Rituximab: p<0.0005; Rituximab vs. BNP2×4: Not significant; Rituximab vs. BNP2×8: p<0.0003) (Figure 5).

The organs and masses recovered from tumor-developing mice (three months after the treatment) were analyzed by H&E in order to confirm the development of BL model. Necrotic/apoptotic areas were still present in mice from groups 5 and 6, in comparison with BNP1-treated animals (Figure S4).

## Discussion

The use of Ab-coated nanoparticles represents a new strategy to target only tumor cells with high-dose chemotherapy, even in the context of an adverse genetic profile. In this contribution, we characterized both *in vitro* and *in vivo* the effects of a new kind of biodegradable nanoparticles coated with the anti-CD20 chimeric antibody Rituximab, and loaded with CLB and HCQ.

CLB, an alkylating agent, has been in use for decades to treat hematological malignancies [33]. This drug is given orally, but causes problems because the rate of drug absorption into the bloodstream can vary significantly from patient to patient [34]. Also, most B cell malignancies will become resistant to this agent at some point no matter whether it is used at increasing doses, or within more aggressive regimens. In resistant situations, it could be important to have a therapeutic system for a better delivery of high amounts of drugs specifically inside B malignant cells in order to circumvent genetically driven tumor mechanisms of resistance.

The combination of an elevated concentration of CLB intracellularly with another kind of pro-apoptotic drug not dependent on surviving genes, could not only enhance their respective cancer killing activities but perhaps make a resistant lymphoma cell sensitive again. HCQ [35] has demonstrated an interesting pro-apoptotic effect. Its anti-neoplastic properties *in vitro* depend on its concentration, but this cannot routinely be obtained *in vivo* by the usual oral route of administration [36], [37]. Its capacity to block the fusion between autophagosomes and lysosome was clearly demonstrated [38], inducing cell cytotoxicity in a p53-independent manner.

BJAB is a well characterized BL cell line, mutated in p53 [39] and expressing high levels of CD20 expression [40]. As others p53 mutated cells, like Raji, BJAB is quite resistant to CLB and other classical chemotherapeutic agents such as anthracyclines and purine analogues; in fact, high doses of CLB were able to induce only 38% of cell cytotoxicity, while fludarabine and doxorubicin kill 42% and 37% of cells, respectively. However, in our study they appeared more susceptible to HCQ (82% of killing). Combining HCQ with other drugs could probably be synergistic, especially for those patients in an already resistant disease state, or with bad prognosis gene mutations, as already described [41]. Our data indicates that only CLB has an additive effect with HCQ, causing 92% of killing. Moreover, side effects induced by these drugs are well described in the literature, [42], [43] and were also evident in our experiments in healthy mice.

These issues were addressed by including these drugs in BNPs produced from biocompatible and biodegradable materials. Receptor-targeted nanoparticles like the ones presented here (200–300 nm) are viewed as good drug carriers: they can transport large amounts of therapeutic agents while having a prolonged circulation time, as well as a very selective tumor penetration when coated with monoclonal antibodies such as the anti-CD20 Rituximab. These nanoparticles (BNP2) can release enough amounts of drugs inside the cancer cells and in this way, overcome multidrug resistance (MDR) mechanisms which are over-expressed in many B-cell disorders [44]. In vitro, we have also documented a cytotoxic effect induced by the penetration of BNP3 in tumor B cells, but their efficacy were not confirmed in vivo probably due to the effect of the blood flow, or the reduced residence time of the untargeted nanoparticles in the tumor microenvironment.

HCQ-CLB-loaded nanoparticles demonstrated their ability to selectively target CD20-positive cells in vivo, resulting in a very promising toxicological profile in healthy mice. The presence of anti-CD20 antibody targets BNPs on the surface of cells expressing this antigen, and induces their preferential penetration in vivo. Confocal and electron microscopy studies showed the interaction between anti-CD20 nanoparticles and the cell membrane. Moreover, BNPs were localized into the cell cytoplasma. Intracellular trafficking of nanoscale objects is regulated by cell membranes through interactions that are currently under intense investigation. These materials are typically internalized by cells into membrane-bound endosomes. Other bio-macromolecules can penetrate or fuse with cell membranes without causing any significant membrane disruption. Some nanoscale objects pass through cell membranes by generating transient holes, a process associated with cytotoxicity [45], [46], [47]. BNPs seem to be internalized outside endosomes, and accumulate in the cytoplasma.

The cytotoxic effect caused by the degradation of the particles and the subsequent release of the two drugs was apparent by analyzing apoptosis just after 16 hours of incubation of lymphoma cells with BNP2. Autophagy is a lysosome-mediated intracellular trafficking pathway often up-regulated to support tumor cell survival [48]. Primary CLL cells, for example, express the critical components of the autophagy machinery, which could be robustly activated or blocked in these cells by commonly reported stimuli like HCQ [30]. LC3 and p62 activations are markers of this process. BNP2 treated cells showed an accumulation of proteins that demonstrate the blocking of autophagolysosome formation, and may partially explain tumor cell cytotoxicity induced by HCQ/CLB-loaded BNPs. In 48 hours, all cancer B-cells were killed.

The loading of Rituximab in BNPs is very low and it is not sufficient to induce apoptosis as shown by incubating BJAB cells with BNP1. The ability of Rituximab to activate the complement system (one of its effector systems) is dependent on the amount of Ab bound to the tumor cell and, as a consequence, to the amount of CD20 on the cell surface [49], [50], [51], [52], [53], [54]. For this reason, we compared the cytotoxic effect of Rituximab and BNP2 in a population of BJAB cells and primary cells purified from five CLL patients expressing a low amount of the tumor-associated antigen. Our data indicate that BNP2 were able to kill cells expressing very low amounts of CD20, where Rituximab was almost ineffective.

BJAB cells were able to develop a human/mouse model of lymphoma in SCID mice. A characteristic feature that this model shares with human aggressive lymphomas, like BL, is the early multi-organ involvement that starts with the formation of a tumor mass. The liver was the main target of the tumor cells outside the peritoneum, and it was involved in nearly all the animals analyzed. This is compatible with the concept of preferential diffusion of the cancer cells into the liver, even though invasion may be initiated by contiguity with the tumor mass as it may occur also in the spleen and, to a much lower extent, in the kidney. Despite the fact that the cells were injected via the intraperitoneal route rather than intravenously, the finding that the bone marrow was infiltrated in approximately 80% of mice provides compelling evidence for the ability of tumor cells to colonize tissues that are rather distant from the primary implantation site, while still maintaining the same morphology and phenotype of the cells in the original tumor mass.

The animal model of BL with multi-organ involvement proved to be a valid preclinical tool to evaluate the anti-tumor effect of BNPs therapy. This is particularly important because the treatment of aggressive lymphoma is currently based on the use of dose-intensive polychemotherapy protocols to eradicate the high tumor bulk [55].

The BNP2 particles demonstrated for the first time their ability to target human tumor B-cells *in vivo*, but also their potential efficacy in tumor bearing mice, showing the binding of fluorescent-antiCD20-BNPs and the evident necrotic/apoptotic areas in the tumor mass 7 days after being injected i.p. The therapeutic protocols used in this study were derived from toxicological data obtained with free HCQ+CLB, because BNPs containing the same amount of drugs never showed tissue damage in healthy animals. Our aim was to mimic the clinical situation i.e., administering the first dose of BNPs on day four after cell injection, when the neoplastic cells were already disseminated, as shown by optical imaging and confocal microscope analysis.

The BNP2 particles were very effective in prolonging the survival of tumor-bearing mice in our model system. Four injections of BNP2 containing 400 μg of HCQ+CLB increase mice survival by about 15% with respect to the same amount of free drug or a standard treatment with Rituximab[29] but without any side effects. Moreover, 8 injections of BNP2 containing 400 μg of HCQ+CLB were able to increase survival to 100% of mice. Ninety percent of animals survived for four months and were disease-free after immunohistochemical analysis. This amount of HCQ+CLB cannot be used as free drugs because of its toxicity, showing again the capacity of BNPs to guarantee the use of high doses of chemotherapeutic drugs while avoiding side effects.

In conclusion, the results of the present study demonstrate that anti-CD20 nanoparticles containing HCQ+CLB can be effective as a single agent in controlling a disseminated model of aggressive lymphoma. It also provides a rationale for adopting this therapeutic approach for the treatment of other B-cell disorders with BNP2 or different types of tumors, using other monoclonal antibodies to specifically deliver cytotoxic agent-loaded nanoparticles in cancer cells.

## Supporting Information

Figure S1 **Characteristics of BNPs used in the experiments.**
(TIF)

Figure S2 **Characterization of Burkitt model in SCID mice.** Labeled BJAB (2×10^6^ cells) were injected i.p. in SCID mice and specific staining of labeled-cells in tissues collected from untreated animals at day 7 was documented. Serial stacks were z-projected using average algorithm. The tissue auto-fluorescence to laser 488 nm is visible in green fluorescence. Objective Plan Apo 20X was used to achieve a low magnification capable of better describing the tissue environment. Original magnification 200×.
(TIF)

Figure S3 **Characterization of Burkitt model in SCID mice.** Liver is infiltrated by neoplastic cells, which show a cohesive and diffuse growth pattern (A) and the same immunophenotypic profile observed in primary masses: anti-CD20 (B) and anti-Bcl-6 (C) (immunostainings, strept-ABC method). D) Foci of neoplastic lymphoid cells can be detected in the bone marrow and highlighted by anti-human-CD45 immunostaining (inset). Original magnification 200×.
(TIF)

Figure S4 **Effect of BNP2 in tumor mass of lymphoma-bearing mice.** SCID mice received 2×10^6^ BJAB cells i.p. and BNP1 or BNP2 (80 μL for 4 times) were injected i.p. from day 4. Tumor mass were collected at necroscopy and analyzed by H&E to detect necrotic/apoptotic areas.
(TIF)
