# Correction: New Potential Therapeutic Approach for the Treatment of B-Cell Malignancies Using Chlorambucil/Hydroxychloroquine-Loaded Anti-CD20 Nanoparticles

**DOI:** 10.1371/annotation/55af21b1-e916-4928-bba3-bf76a7abfc9d

**Published:** 2013-11-05

**Authors:** Nelly Mezzaroba, Sonia Zorzet, Erika Secco, Stefania Biffi, Claudio Tripodo, Marco Calvaruso, Ramiro Mendoza-Maldonado, Sara Capolla, Marilena Granzotto, Ruben Spretz, Gustavo Larsen, Sandra Noriega, Marianna Lucafò, Eduardo Mansilla, Chiara Garrovo, Gustavo H. Marín, Gabriele Baj, Valter Gattei, Gabriele Pozzato, Luis Núñez, Paolo Macor

The affiliation of author Chiara Garrovo was incorrect.

The correct affiliation for this author is: Institute for Maternal and Child Health – IRCCS “Burlo Garofolo”, Trieste, Italy 

