# Supplementary figures and images for: New Potential Therapeutic Approach for the Treatment of B-Cell Malignancies Using Chlorambucil/Hydroxychloroquine-Loaded Anti-CD20 Nanoparticles
Source: PLoS One. 2013 Sep 30;8(9):e74216. doi: 10.1371/journal.pone.0074216 (PMC3787049; doi:10.1371/journal.pone.0074216)

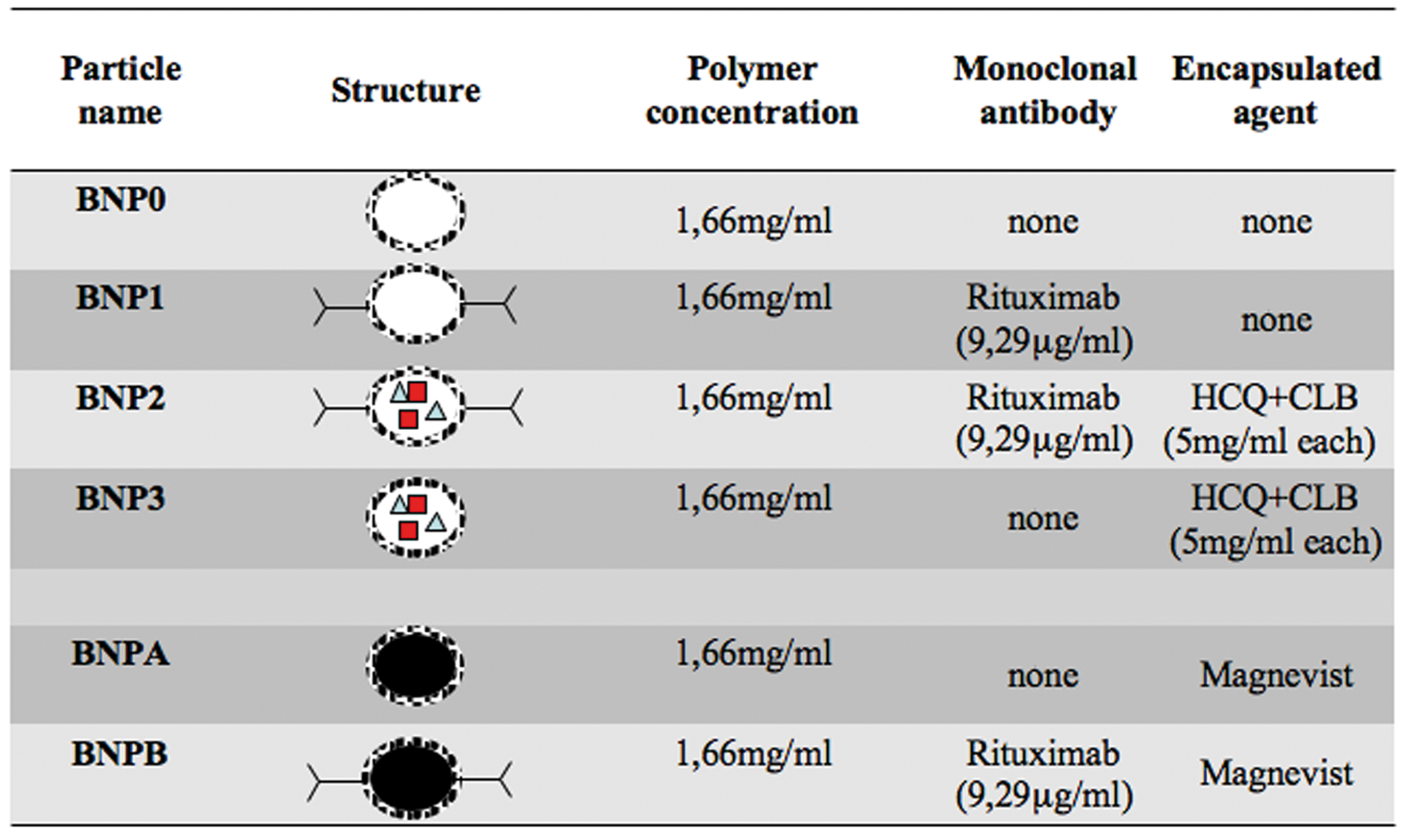

Supplement: Figure S1 — Characteristics of BNPs used in the experiments. (TIF) [file pone.0074216.s001.tif]

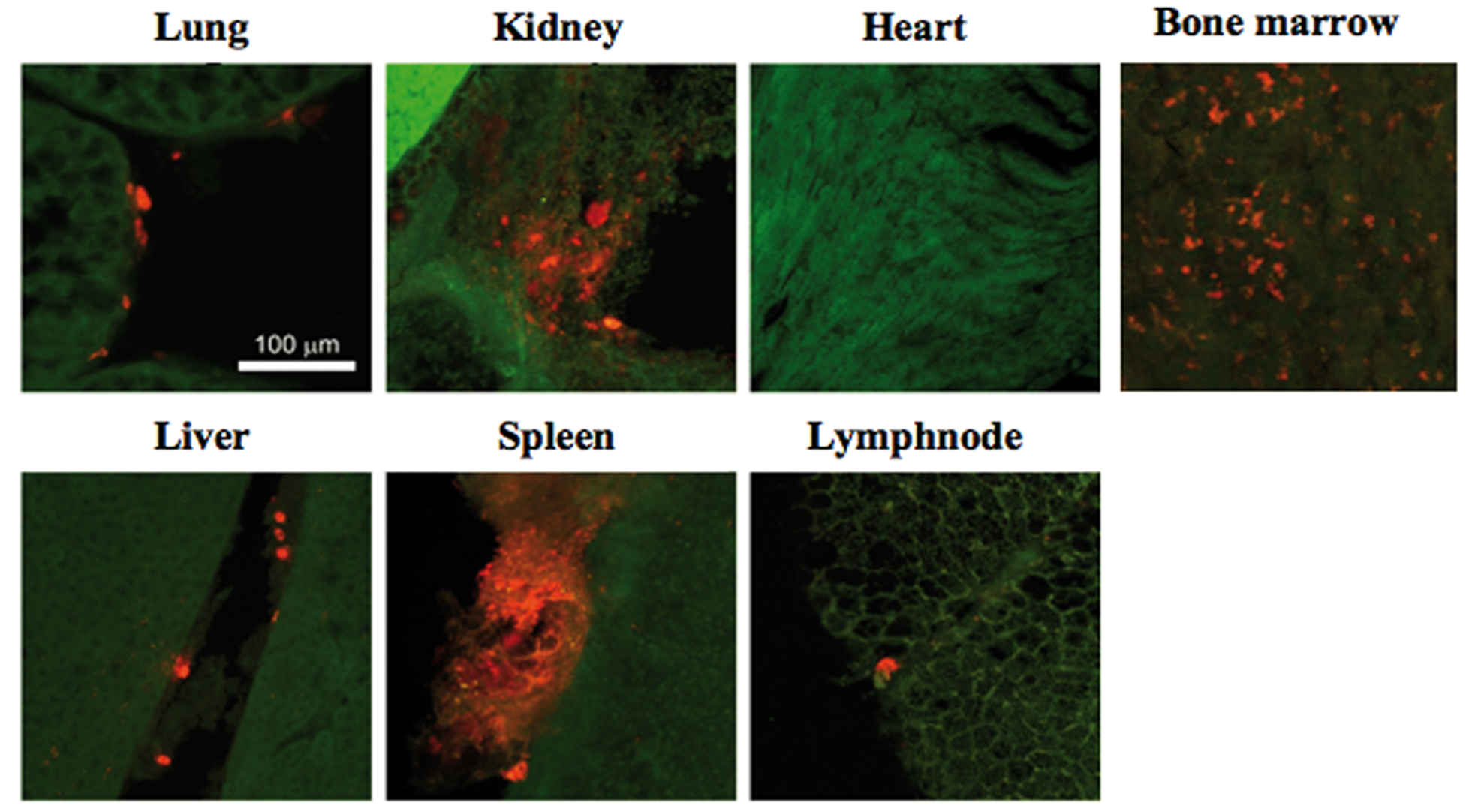

Supplement: Figure S2 — Characterization of Burkitt model in SCID mice. Labeled BJAB (2×10^6^ cells) were injected i.p. in SCID mice and specific staining of labeled-cells in tissues collected from untreated animals at day 7 was documented. Serial stacks were z-projected using average algorithm. The tissue auto-fluorescence to laser 488 nm is visible in green fluorescence. Objective Plan Apo 20X was used to achieve a low magnification capable of better describing the tissue environment. Original magnification 200×. (TIF) [file pone.0074216.s002.tif]

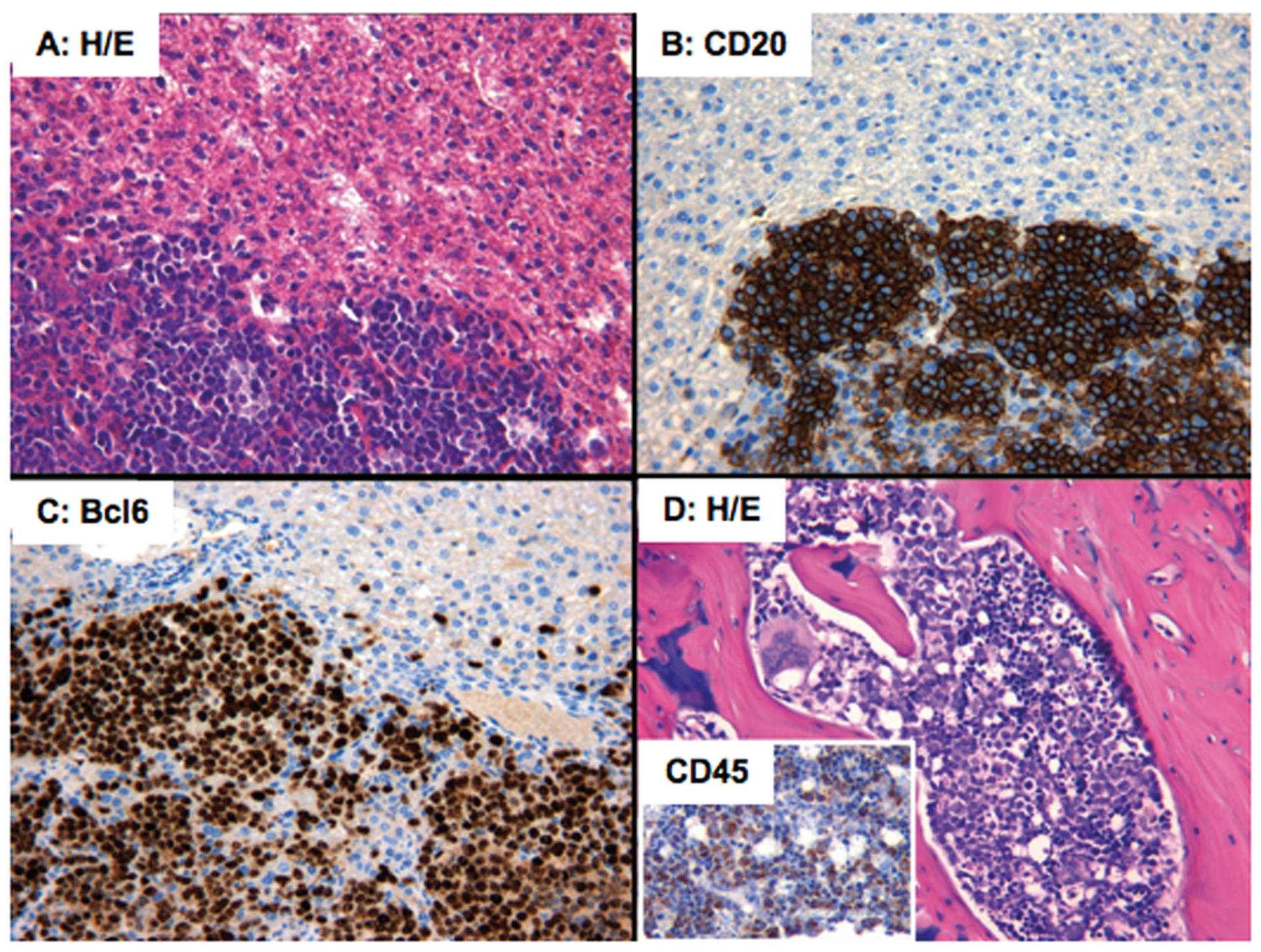

Supplement: Figure S3 — Characterization of Burkitt model in SCID mice. Liver is infiltrated by neoplastic cells, which show a cohesive and diffuse growth pattern (A) and the same immunophenotypic profile observed in primary masses: anti-CD20 (B) and anti-Bcl-6 (C) (immunostainings, strept-ABC method). D) Foci of neoplastic lymphoid cells can be detected in the bone marrow and highlighted by anti-human-CD45 immunostaining (inset). Original magnification 200×. (TIF) [file pone.0074216.s003.tif]

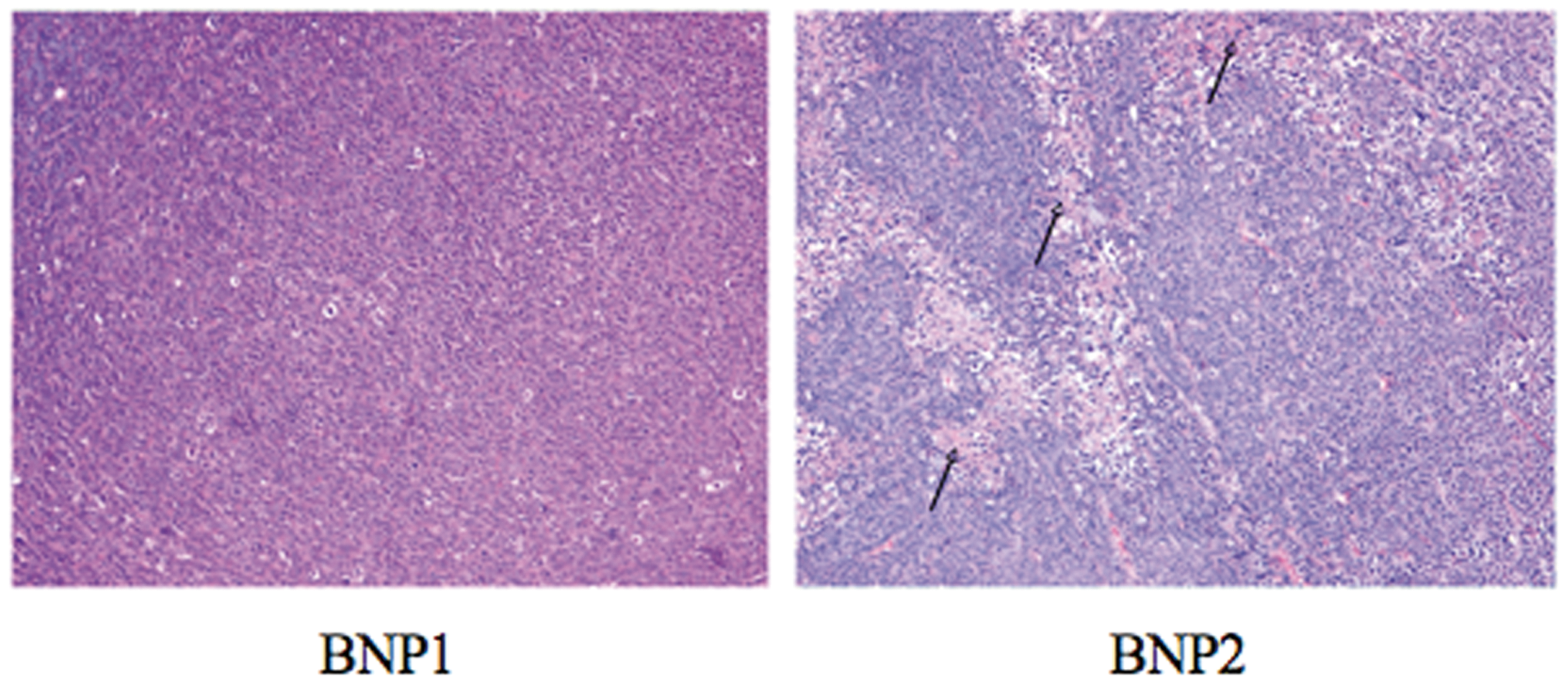

Supplement: Figure S4 — Effect of BNP2 in tumor mass of lymphoma-bearing mice. SCID mice received 2×10^6^ BJAB cells i.p. and BNP1 or BNP2 (80 μL for 4 times) were injected i.p. from day 4. Tumor mass were collected at necroscopy and analyzed by H&E to detect necrotic/apoptotic areas. (TIF) [file pone.0074216.s004.tif]
